# Enhanced Activation of Memory, but Not Naïve, B Cells in Chronic Hepatitis C Virus-Infected Patients with Cryoglobulinemia and Advanced Liver Fibrosis

**DOI:** 10.1371/journal.pone.0068308

**Published:** 2013-06-28

**Authors:** Deanna M. Santer, Mang M. Ma, Darren Hockman, Abdolamir Landi, D. Lorne J. Tyrrell, Michael Houghton

**Affiliations:** 1 Li Ka Shing Institute of Virology, University of Alberta, Edmonton, Alberta, Canada; 2 Department of Medical Microbiology and Immunology, University of Alberta, Edmonton, Alberta, Canada; 3 Department of Gastroenterology, University of Alberta, Edmonton, Alberta, Canada; Karolinska Institutet, Sweden

## Abstract

Mixed cryoglobulinemia is the most common extrahepatic disease manifestation of chronic hepatitis C virus (HCV) infection, where immunoglobulins precipitate at low temperatures and cause symptoms such as vasculitis, glomerulonephritis and arthralgia. HCV-associated cryoglobulinemia is also strongly linked with the development of B cell non-Hodgkin lymphoma. Abnormal B cell function in HCV infections can lead to the formation of HCV cryoglobulin complexes that usually comprise monoclonal rheumatoid factor and HCV-specific immune complexes. The aim of this study was to characterize the activation phenotype of B cells from patients with chronic HCV infection in comparison to healthy controls using flow cytometry. In addition, we determined how the activation status varies depending on the presence of cryoglobulinemia and advanced liver fibrosis. We found that only memory B cells, not naïve cells, were significantly activated in chronic HCV infection when compared with healthy controls. We also identified markers of memory B cell activation that were specific for HCV patients with cryoglobulinemia (CD86, CD71, HLA-DR) and advanced liver disease (CD86). Our results demonstrate that HCV infection has differential effects on B cells depending on the severity of hepatic and extrahepatic disease.

## Introduction

Approximately ∼170–200 million people around the world are infected with the hepatitis C virus (HCV). 70–80% of patients develop a chronic infection which can lead to liver fibrosis and cirrhosis and an increased risk for developing hepatocellular carcinoma (HCC) [1]. Extrahepatic manifestations also occur in patients with chronic HCV infection including kidney and skin disease, with the most common extrahepatic manifestation being mixed cryoglobulinemia [2].

Cryoglobulins are immunoglobulin complexes that precipitate at temperatures less than 37°C and redissolve upon rewarming. Cryoglobulins are classified into 3 types based on their immunoglobulin (Ig) makeup: I, II and III [3]. Type I cryoglobulins consist of monoclonal IgG or IgM antibodies and are not typically associated with HCV and are usually found in patients with lymphoid tumors. Type II cryoglobulins typically consist of monoclonal IgM with enriched rheumatoid factor activity and polyclonal IgG, whereas type III cryoglobulins differ in that all Igs are polyclonal. Both type II and type III are considered ‘mixed cryoglobulinemia’ and were initially discovered to be associated with HCV infection in 1991, shortly after the discovery of HCV in 1989 [4], [5]. In addition to IgG and IgM, the cryoprecipitate contains HCV antigens (especially the nucleocapsid antigen), an abundance of HCV RNA and complement proteins such as C1q [6], [7]. Cryoglobulins can be detected in up to 60% of HCV patients, but only 5–20% of patients present clinical signs of cryoglobulinemia syndrome with type II cryoglobulins predominating in HCV [6], [8], [9]. The three most common symptoms of cryoglobulinemia syndrome are purpura, arthralgia, and weakness, and less commonly glomerulonephritis, skin ulcers and diffuse vasculitis may be present [10]. Patients with cryoglobulinemia also have an increased incidence of liver cirrhosis with an odds ratio of 4.87 [6], [11].

The relationship between HCV and mixed cryoglobulinemia was identified more than 20 years ago [5], but the mechanism by which HCV causes B cell proliferation/activation is still not understood. Understanding this mechanism is especially important because a subset of individuals with HCV infection and type II cryoglobulins will develop B cell non-Hodgkin lymphoma (B-NHL) [12]. Recent work by Visentini et al. [13], Charles et al. [14], [15] and Terrier et al. [16] have elegantly outlined the characteristics of a subset of clonally-expanded CD21^−/low^ IgM^+^CD27^+^ B cells in cryoglobulinemia which are enriched in V_H_1–69 and V_k_3–20 gene segments that code for a rheumatoid factor typically of the Wa idiotype [17]. This B cell subset has been found to be exhausted and more prone to undergo apoptosis and most recently, gene pathways were identified that could regulate the B cell dysfunction observed (eg. [13], [14]. Recently, three publications studied the B cell phenotype in chronic HCV infection with varying results [18], [19], [20]. Currently there are 3 proposed mechanisms for how HCV activates B cells: 1) via HCV E2 envelope glycoprotein binding it’s CD81 tetraspanin receptor, 2) via HCV-B cell receptor (BCR) interactions and/or 3) via HCV infection and replication in B cells.

The purpose of this study was to determine if changes in terms of the numbers and activation status of total B cells and B cell subsets exist in patients with chronic HCV infection compared to healthy controls. Secondly, we sought to compare the B cell phenotype in HCV patients with or without cryoglobulinemia and those with or without advanced liver disease, both of which were not extensively studied previously. In summary, we found that while the percentages and absolute numbers of B cells were not strikingly different during chronic HCV infection, memory B cell, but not naïve B cell, activation was clearly evident in HCV patients’ peripheral blood. Importantly, we identified three activation markers that were significantly elevated in cryoglobulin-positive HCV patients compared to cryoglobulin-negative HCV patients (CD86, HLA-DR and CD71). In addition, we found that CD86 was specifically upregulated on memory B cells from HCV patients with advanced liver disease. Our results demonstrate that memory B cells are preferentially activated in chronic HCV infection and that the presence of cryoglobulins and/or fibrosis can enhance this phenomenon.

## Materials and Methods

### Ethics Statement

All donors gave written informed consent. Study protocols were approved by the Health Research Ethics Board at the University of Alberta, Edmonton, Alberta, Canada.

### Study Subjects

We enrolled 54 chronically infected HCV patients and 50 healthy controls for this study over a period of 7 months. All HCV patients were HCV Ab+, HIV Ab-, hepatitis B surface antigen (HBsAg)- and HCV RNA+, whereas all healthy control subjects were HCV Ab-, HIV Ab- and HBsAg-. No subjects were currently receiving or had received interferon or immunosuppressive therapy within 6 months of the enrollment date. Additional exclusion criteria included: recent illness and/or vaccine within 4 weeks prior to the blood draw, presence of autoimmune disease unrelated to HCV and treatment with any anti-inflammatory medications. The patients and controls’ characteristics are summarized in Table 1.

**Table 1:** Clinical characteristics of HCV+ patients and healthy controls in study.

	HCV+ patients	Healthy Controls
Number of subjects	54	50
Sex, M/F	29/25	27/23
Age, mean years +/− SD	52.4+/−10.5	50.3+/−8.2
ALT, median U/L (range)	85.5 (20–386)	26.0 (11–49)
GGT, median U/L	54.5 (5–593)	12 (5–66)
HCV viral load, median IU/ml (range)	3.92×10^6^ (1.7×10^5^–3.95×10^7^)	N/A
Viral Genotype		
1	41	N/A
2	3	
3	8	
4	2	
Fibrosis stage (0–4)*	Known for 53 patients: 5 (F0), 14 (F1), 8 (F2), 10 (F3), 16 (F4)	N/A
Cryoglobulinemia (Type)	20 (16 type II, 4 type III)	None
History of treatment (tx)	30 tx naïve, 24 failed IFN/Ribavirin tx	N/A

Abbreviations: N/A, not applicable, ALT, alanine aminotransferase (normal values = <50), GGT, gamma-glutamyl transpeptidase (normal levels = <70 (males), <55 (females)), IU, International units, IFN, interferon.

* determined from biopsy or Fibroscan.

### Clinical Tests

Serum alanine aminotransferase (ALT), gamma-glutamyl transpeptidase (GGT), alpha-fetoprotein (AFP) plus complete blood count with differential and cryoglobulin testing was done on all patients and controls at the time of study (exception: AFP was only run for 42 patients and GGT was done for 48 patients and 47 healthy controls). All healthy controls’ results were within normal ranges and all were cryoglobulin negative. For cryoglobulin testing, blood collected in red top BD Vacutainer® serum tubes (BD Biosciences, Inc.) was placed in a 37°C water bath immediately for 30 min−1 hr and centrifuged for 10 min at 1100 g at 37°C to collect serum which was promptly separated and stored at −80°C or put at 4°C for 3–4 days for cryoglobulin quantification and typing (typing done by DynaLIFE_Dx_). All positive cryoglobulin precipitates (cryoprecipitate) were able to be re-dissolved at 37°C. The percent of total volume (cryocrit) was determined upon centrifuging serum on day 3–4 at 4°C at 2000 rpm for 10 min as described [21]. For 53/54 patients the stage of liver fibrosis was known as was determined by a recent biopsy or by transient elastography (Fibroscan). The fibrosis scores were noted from normal (0) – cirrhosis (4) as per the METAVIR scoring system [22].

### Determination of HCV Serum Titers

RNA was isolated from 50 µl of patient serum with a Roche High Pure Viral Nucleic Acid Kit according to the manufacturer's instructions (Version 15.0, Roche Diagnostics, Inc.). HCV RNA titers were determined using an in-house qRT-PCR assay that was normalized to select titers determined via COBAS® Ampliprep/COBAS® TaqMan® HCV Test (Roche Molecular Systems, Inc.) to give results in standardized international units (IU)/ml. Isolated RNA was dried in a speed-vacuum (1000 rpm, 1.5 hr, 60°C) and resuspended in water for reverse-transcription using Thermoscript™ RNase H (Life Technologies Corp.) with the following HCV-specific primer: 5′-GTGTTTCTTTTGGTTTTTCTTTGAGGTTTAGG. qRT-PCR was performed using a real-time PCR system (model CFX96, Bio-Rad Laboratories, Inc.) and TaqMan® chemistry, with measurements in triplicate. We used 6-FAM-CACGGTCTACGAGACCTCCCGGGGCAC-TAMRA as the HCV-specific detection probe and a primer set detecting the conserved 5′-untranslated region of HCV (Fwd: 5′-TCTGCGGAACCGGTGAGTA; Rev: 5′- GTGTTTCTTTTGGTTTTTCTTTGAGGTTTAGG). All primers were synthesized by Integrated DNA Technologies, Inc. Our detection limit was 25 RNA copies/ml. For absolute quantification, we created a standard curve of known dilutions of a plasmid containing the sequence for HCV JFH-1 alongside a positive control (HCV serum quantified via the COBAS® kit) and negative control (water and RT reaction alone).

### Blood Collection and Processing

A total of 20–30 ml of blood per patient/control was collected into BD Vacutainer® sodium heparin tubes (BD Biosciences, Inc.). Peripheral blood mononuclear cells (PBMCs) were isolated by ficoll gradient within 2 hrs of blood draw and resuspended in RPMI 1640 media with 10% heat inactivated FBS, 1% penicillin/streptomycin, 10 mM HEPES, 1X non-essential amino acids, 1 mM sodium pyruvate and 2 mM GlutaMAX (all from Life Technologies, Corp.).

### Flow Cytometry

**Figure 1:**
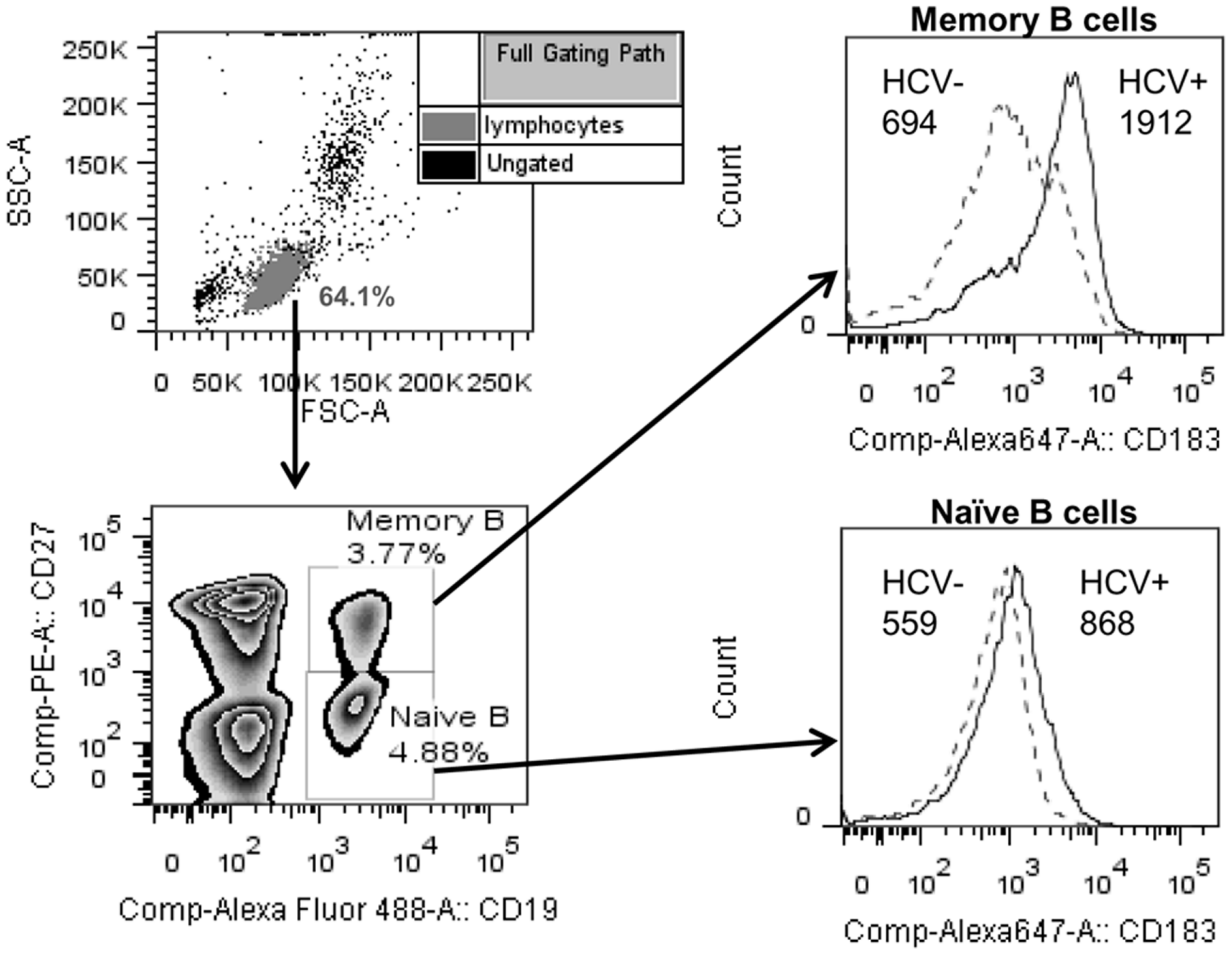
Gating strategy for flow cytometry analysis of memory and naïve B cell activation. Lymphocytes were gated by FSC/SSC properties and memory B cells (CD19+CD27+) and naïve B cells (CD19+CD27−) were analyzed within the lymphocyte gate for the expression of 6 different markers described in the Materials and Methods. Histograms show a representative example of the expression of CD183 (numbers represent geometric mean fluorescent intensity) on memory and naïve B cells from a healthy control (HCV-) and chronic HCV patient (HCV+).

Fresh PBMCs (7.5×10^5^) were analyzed by flow cytometry to look at B cell activation after gating on naïve (CD19+CD27−) and memory (CD19+CD27+) B cells. The following mouse mAbs were used with the corresponding mouse IgG isotypes: anti- CD86 Alexa Fluor 647 (clone IT2.2), CD69 PerCP/Cy5.5 (clone FN50), HLA-DR Alexa Fluor 647 (clone L243), CD40 PerCP/Cy5.5 (clone 5C3), CD71 APC (clone OKT9), CD183 (CXCR3) Alexa Fluor 647 (clone TG1/CXCR3), CD19 Alexa Fluor 488 (clone HIB19), CD5 PerCP/Cy5.5 (clone UCHT2), CD27 PE (clone O323). All antibodies above were from Biolegend, Inc. except CD71 APC which was from eBioscience, Inc. All flow cytometry antibodies were titrated before use to determine optimal staining conditions. Cells were incubated with combinations of the above antibodies for 25 min at 4°C, washed with PBS +1% BSA and fixed in 2% paraformaldehyde (in PBS) for 15 min at room temperature before washing and resuspending in PBS +1% BSA until data acquisition on a FACSCanto II flow cytometer (BD Biosciences, Inc.). Data was analyzed using FlowJo software (Tree Star, Inc.). Geometric mean fluorescent intensities and % positive values (determined with mouse isotype controls) were compared for each activation marker between groups for each subset of B cells. Our gating strategy and example histograms are shown in Figure 1.

### Statistical Analysis

Statistical significance between groups was determined by Mann-Whitney test or unpaired t test, where appropriate. Correlations between parameters were assessed using the Spearman or Pearson correlation analysis. A P-value <0.05 was considered significant. Graphs and statistical analyses were performed using Prism software (v 5, Graphpad Software, Inc.).

## Results

### Clinical Characteristics of Study Participants

A total of 54 chronically infected HCV+ patients and 50 age and sex matched healthy controls were enrolled for this study (summarized in Table 1). As expected, the majority (41/54) of patients were infected with genotype 1 HCV (21/41, 1A; 12/41, 1B; 5/41 A or B; 2/41, 1A +1B; 1/41, 1A +1C), whereas 3, 8 and 2 patients were infected with genotypes 2, 3 or 4, respectively. We tested each individual as they came into the clinic for the presence of cryoglobulins in the form of cryoprecipitates (cryo) and found that 20/54 (37%) of the HCV patients were cryo+. Only 6/20 cryo+ patients were previously diagnosed to have cryoglobulinemia before entering our study. Of the 20 patients, 16 had type II cryo, whereas 4 had type III cryo. A total of 11/20 patients were either currently experiencing symptoms or had experienced symptoms related to cryoglobulinemia in the past. The symptoms ranged from vasculitis (legs) (7 patients) to joint pain (1 patient) to glomerulonephritis (3 patients). Cryoglobulins were not detected in any of the 50 healthy controls and all their clinical tests were within the normal range.

### Chronically-infected HCV Patients have Slightly Elevated Percentages of Peripheral B Cells, but no Changes in B cell Subset Frequency

**Figure 2:**
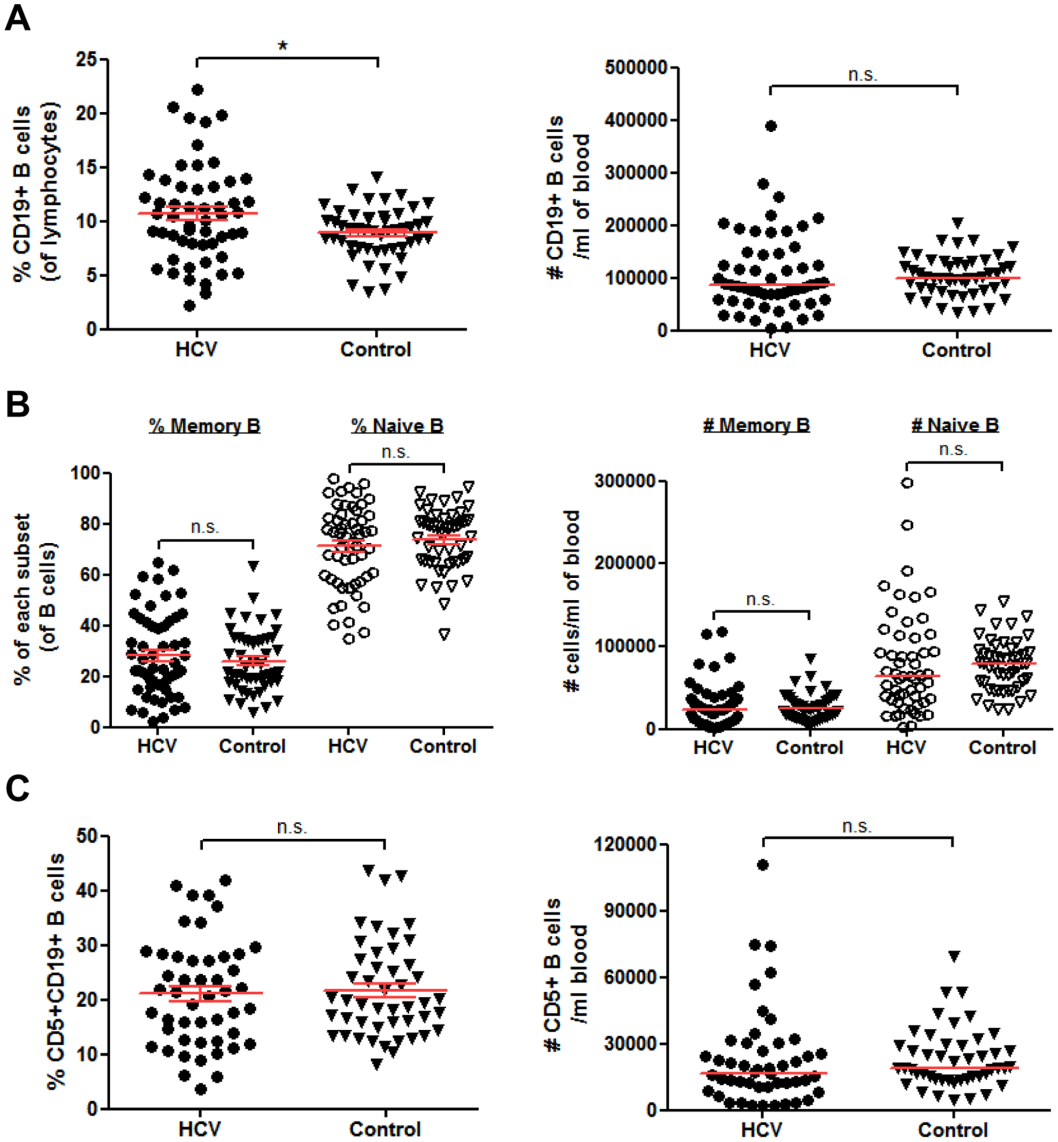
B cell subset frequencies are unchanged in HCV patients compared to controls. (**A–C**) 7.5×10^5^ freshly isolated PBMCs from chronically infected HCV patients (n = 54) or healthy controls (n = 50) were incubated with fluorescently labeled antibodies to CD19, CD27 and CD5 for multi-color flow cytometry analysis. (**A**) The total percent of CD19+ B cells within the lymphocyte gate and number of B cells per ml of blood based on our isolations. (**B**) The percent of memory (CD19+CD27+) and naïve (CD19+CD27−) and number of each subset per ml of blood. **(C)** The percent and number per ml of CD5+CD19+ B cells. Horizontal lines on graphs represent means +/− SEM (percentages) or median (number of cells/ml) values. In (C) only 48 HCV and 47 healthy control samples were tested for CD5. *, P<0.05; n.s., not significant.

We first asked if the total numbers of CD19+ B cells, memory and naïve subsets and their proportions expressed as percentage of total lymphocytes were altered in HCV patients compared to healthy controls. In our cohort, the proportion of CD19+ B cells as the percent of total lymphocytes was significantly elevated in HCV patients, but the number of B cells per ml of blood was not significantly changed (Figure 2A). The number of memory and naïve B cells and their proportion expressed as percent of total B cells were not significantly different between HCV patients and controls (Figure 2B). We also calculated the proportion and number of CD5+ CD19+ B cells (also known as B1 cells) as this subset is associated with various autoimmune and lymphoproliferative disorders [23], and was shown to be expanded in a cohort of HCV patients [24]. The CD5+CD19+ B cell population was not significantly changed in our cohort of HCV patients compared to healthy controls (Figure 2C). Therefore, only the proportion of CD19+ B cells within the lymphocyte population was significantly different during a chronic HCV infection.

### Memory, but not Naïve B cells, are Significantly Activated in Chronic HCV Infection

**Figure 3:**
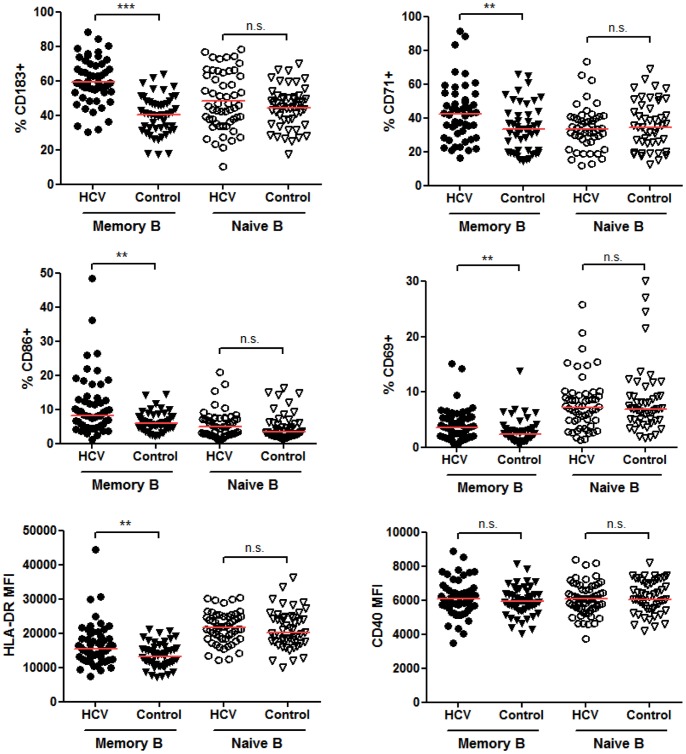
Memory, but not naïve B cells are significantly activated in chronically infected HCV patients. PBMCs were freshly isolated and analyzed by flow cytometry as in Figure 2. Antibodies to CD183, CD71, CD86, CD69, HLA-DR and CD40 were added in combinations with CD19 and CD27 to gate on memory (CD19+CD27+) and naïve (CD19+CD27−) B cells. The percent positive was calculated based on mouse isotype controls and the geometric mean fluorescent intensities (MFI) for each marker were determined. HLA-DR and CD40 are expressed by all B cells therefore only the MFI is shown. Horizontal lines on graphs represent median values. For CD71 analysis, only 48 HCV and 47 healthy control samples were stained. ***, P<0.001, **, P<0.01, *, P<0.05, n.s., not significant.

While the status of B cell activation has been previously examined in patient cohorts from Italy and the United States, the results were not in agreement [18], [19], [20]. Reasons for these differences could have been due to the differences in patient populations or technical differences, especially as it was shown by Rosa et al. [20] that using cryopreserved PBMCs (as opposed to fresh cells) can significantly alter activation marker levels on B cells. We chose to examine 5 markers of B cell activation on freshly isolated PBMCs: CD71, CD86, CD69, HLA-DR, CD40, plus the chemokine receptor CD183 (CXCR3) which was found to be upregulated on HCV patients’ B cells in previous studies [19], [20]. These markers were chosen based on the previous publications above to determine if our results were similar to the other HCV patient cohorts plus we added in HLA-DR and CD40 as additional markers of activation since they have been shown previously to be upregulated in response to stimulation [25]. As shown in Figure 3, the percentage of memory, but not naïve B cells, expressing CD183, CD71, CD86 and CD69 was significantly elevated in chronically infected HCV patients. In addition, the geometric mean fluorescent intensities (MFI) were significantly increased for the above markers plus HLA-DR (MHC II) (Figure 3, Table 2), indicating the relative expression level per cell was increased. CD40 was not upregulated in HCV patient memory or naïve B cells (Table 2). The only marker we examined that was significantly elevated on naïve B cells at the MFI expression level was CD183, although the percentage of naïve B cells expressing CD183 did not change. Levels of CD86 on memory B cells positively correlated with the expression of CD183, HLA-DR, CD71 and CD69 on memory cells indicating that B cells upregulate more than one activation marker during chronic stimulation conditions (P<0.05−P<0.001). Memory CD183 expression levels only correlated with HLA-DR and CD86 (P<0.01). Lastly, the levels of CD86, CD183, CD71, CD69 and HLA-DR on memory B cells significantly correlated with the levels on naïve B cells (P<0.0001). This indicates that although in general naïve B cells were not significantly activated when comparing HCV patients to controls, the HCV patients with the highest activation of memory B cells had a trend towards increased levels of activation markers on naïve B cells.

**Table 2:** Geometric mean fluorescent intensities of each marker on memory and naïve B cells.

	Memory B cells (Geometric MFI*)			Naïve B cells (Geometric MFI)		
	HCV	Control	P-value	HCV	Control	P-value
**CD183**	883.0	520.5	*P<0.0001*	638.5	607.5	*P = 0.033*
**CD71**	543.0	441.0	*P = 0.019*	467.0	452.0	P = 0.988
**CD86**	178.5	141.0	*P<0.0001*	116.0	104.5	P = 0.074
**CD69**	105.5	66.5	*P = 0.016*	56.6	51.0	P = 0.101
**HLA-DR**	15400	13150	*P = 0.0031*	21700	20050	P = 0.5231
**CD40**	6101	5930	P = 0.3689	6077	6153	P = 0.6891
**CD23**	N/A	N/A	N/A	688	852	*P = 0.027*

Abbreviation: MFI, mean fluorescent intensity.

There were no significant correlations or differences seen with any B cell activation marker and the following clinical parameters: serum HCV titer, treatment history, genotype, and age. However, there were significant correlations within the HCV patient population between B cell activation markers and liver function tests for serum ALT, GGT and AFP levels as outlined in Table 3.

**Table 3:** Summary of significant correlations between B cell activation marker expression and liver clinical tests.

ALT			GGT			AFP		
Geo MFI^#^	r	P-value	Geo MFI	r	P-value	Geo MFI	r	P-value
**Memory CD69**	0.3824	0.0043	**Memory CD40**	−0.3771	0.0082	**Memory CD86**	0.4014	0.0084
**Memory CD40**	−0.4929	0.0002	**Naïve CD71**	0.3663	0.0123			
**Naïve CD183**	0.2799	0.04						
**Naïve CD40**	−0.4386	0.0009						
**Naïve CD86**	0.2757	0.0436						

Abbreviations: ALT = alanine aminotransferase, GGT = gamma-glutamyl transpeptidase, AFP = alpha-fetoprotein, Geo MFI = geometric mean fluorescent intensity.

### HCV Patients with Cryoglobulins have Greater Memory B cell Activation Compared to Patients without Cryoglobulins

In a previous study of B cell activation in HCV patients, activation markers were not significantly elevated if cryoglobulins were detected [20]. Since <5–20% of cryo+ patients are symptomatic at a given time point, we tested every patient (and control) for cryoglobulins on the day of enrollment and used this as the basis for scoring patients with cryoglobulinemia rather than clinical symptoms. If this was not done, we would have had included 9 of our cryo+ patients in our HCV cryo- group as those 9 patients had no clinical signs of cryoglobulinemia (cryoglobulin testing is not routinely done in the clinic unless there are symptoms). Our final analysis included 20 cryo+ and 34 cryo- HCV patients. The range of cryocrit, or the amount of cryoglobulin precipitating per volume of serum (See Materials and Methods), varies among patients: typically 1–7%, but it can be as high as >10% [26]. Our cohort of 20 cryo+ patients had an average percent cryocrit of 2.31+/−0.998% (SD).

**Figure 4:**
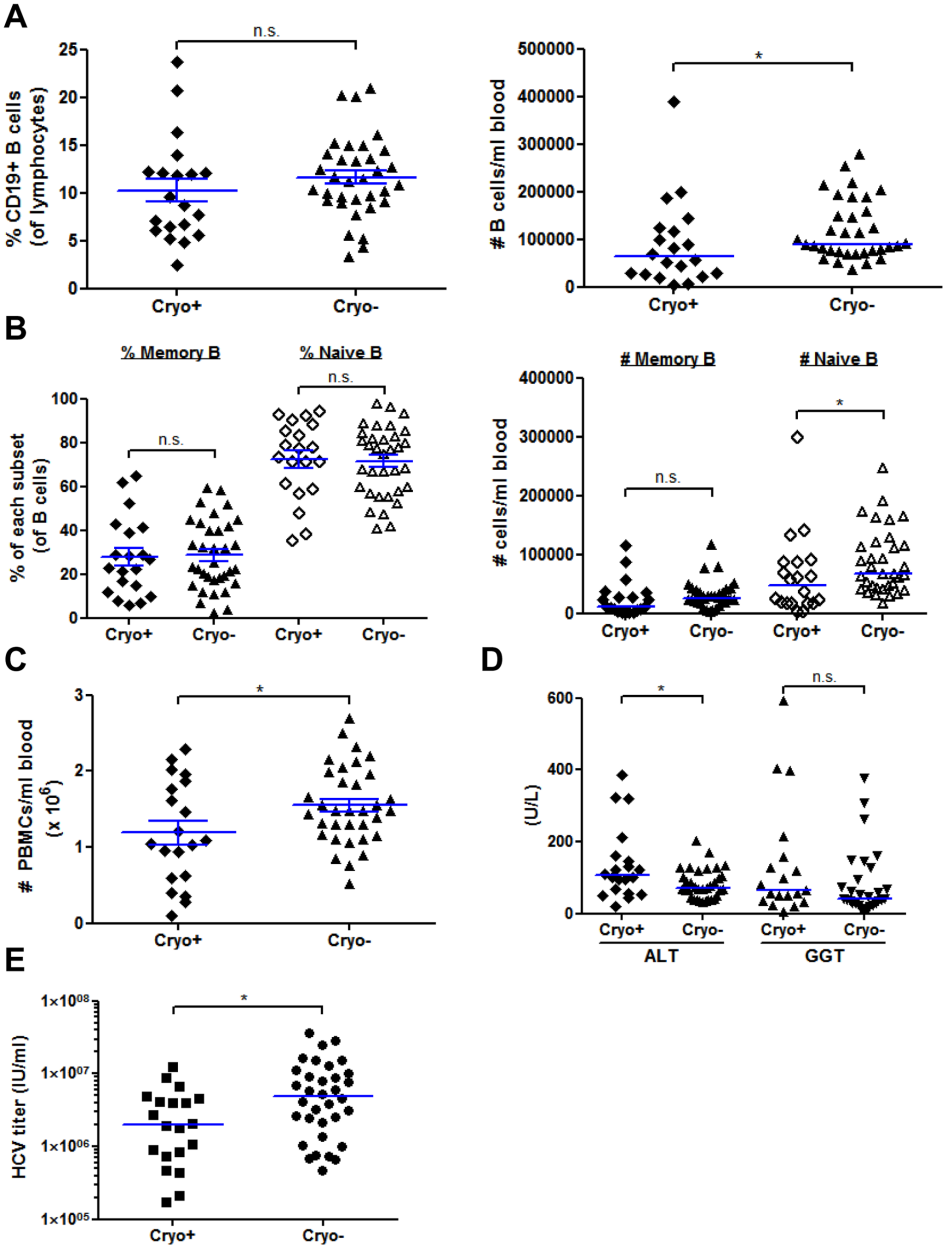
PBMC and B cell quantification and clinical characteristics of cryoglobulin positive versus cryoglobulin negative HCV patients. (**A–B**) PBMCs were freshly isolated and analyzed by flow cytometry as in Figure 2 except that this analysis compares cryoglobulin+ (n = 20, cryo+) and cryoglobulin- (n = 34, cryo-) HCV patients. (**A**) The total percent of CD19+ B cells within the lymphocyte gate and number of B cells per ml of blood based on our isolations. (**B**) The percent of memory (CD19+CD27+) and naïve (CD19+CD27−) and number of each subset per ml of blood. (**C**) The number of total PBMCs isolated per ml of blood. (**D**) The alanine aminotransferase (ALT) and gamma-glutamyl transpeptidase (GGT) levels were measured in the serum in units/L (U/L) for 54 (ALT) or 48 (GGT) HCV patients. (**E**) Serum HCV RNA titers were quantified by qRT-PCR as described in the Materials and Methods for all 54 HCV patients. Horizontal lines on graphs represent mean +/− SEM (A-B, percentages, C) or median (A–B numbers, D, E) values. *, P<0.05, n.s., not significant. IU/ml, international units/ml.

We first analyzed if B cell percentages (percent of total lymphocytes) differ between cryo+ and cryo- patients. We found that the percentage of CD19+ B cells was not significantly changed in patients with cryoglobulins, but the number of B cells per ml of blood was significantly decreased compared to cryo- samples (Figure 4A). In contrast to Oliviero et al. [19], we did not find any differences in B cell memory or naïve subset percentages or numbers (Figure 4B). In addition, the percent of CD19+CD5+ B1 B cells was higher in cryo+ patients, but this trend did not reach statistical significance (P = 0.1492, data not shown). The number of PBMCs isolated per ml of blood was significantly decreased in cryo+ patients (Figure 4C). Among type II cryo+ patients, the differences are more striking with memory B cell numbers significantly decreased (data not shown). Overall white blood cell (WBC) counts were lower in cryo+ patients, but the result was not significant (P = 0.1345). In our cohort, cryo+ patients had significantly higher serum ALT values (P = 0.0316), but not serum GGT values (P = 0.1911) (Figure 4D), and significantly lower HCV RNA serum titers (P = 0.0176) (Figure 4E), although this could be partially due to the underestimation of HCV RNA present in serum if RNA-containing cryoglobulins are present as suggested by Schmidt et al. [21]. After analyzing HCV patients for the presence or absence of symptoms, we found the same significant decrease in the number of naïve B cells/ml of blood and # PBMCs/ml of blood and a significant increase in serum ALT values in patients with a history of symptoms (data not shown, P<0.05).

**Figure 5:**
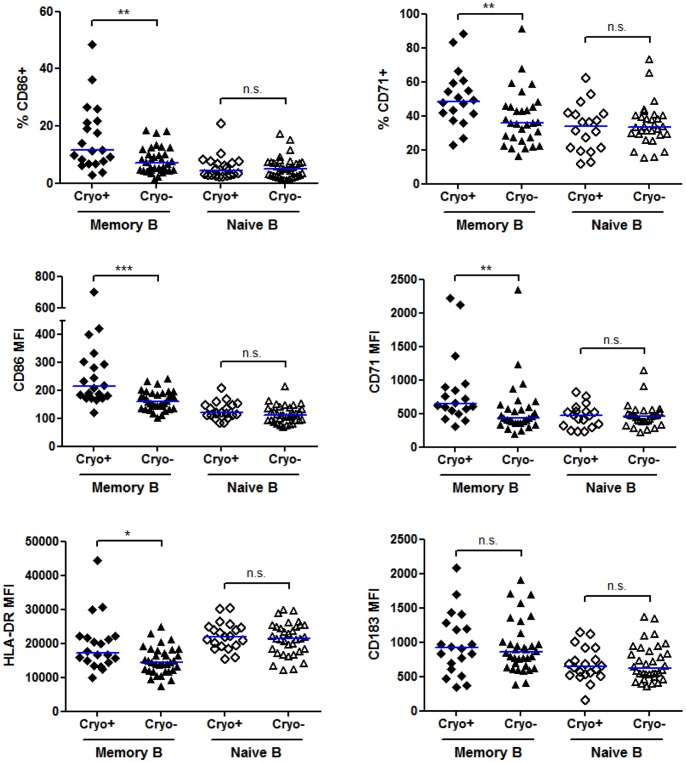
Memory, but not naïve B cells from cryoglobulin positive HCV patients express higher levels of activation markers compared to B cells from cryoglobulin negative HCV patients. PBMCs were freshly isolated and analyzed by flow cytometry as in Figure 3 except that this analysis compares cryoglobulin+ (n = 20, cryo+) and cryoglobulin- (n = 34, cryo-) HCV patients. Antibodies to CD86, CD71, HLA-DR and CD183 were added in combinations with CD19 and CD27 to gate on memory (CD19+CD27+) and naïve (CD19+CD27−) B cells. The percent positive was calculated based on mouse isotype controls and the geometric mean fluorescent intensities (MFI) for each marker were determined. Horizontal lines on graphs represent median values. For CD71 analysis, 18 cryo+ and 30 cryo- HCV samples were stained. ***, P<0.001, **, P<0.01, *, P<0.05, n.s., not significant.

Since studies have not fully examined the B cell activation status in cryo- versus cryo+ HCV patients with or without symptoms, we examined the levels and percentages of activation markers on naïve and memory B cells within these groups. Naïve B cells from cryo+ patients did not have significant changes in any marker studied (Figure 5). In contrast, we found that CD86, CD71 and HLA-DR were significantly upregulated on memory cells in cryo+ patients as compared to cryo- HCV patients, whereas CD40 and CD69 were unchanged (Figure 5 and data not shown). While CD183 was the most dramatically changed in HCV patients versus healthy controls, CD183 levels were not significantly different between cryo+ and cryo- HCV patients (Figure 5). Naïve B cell, but not memory B cell, expression of CD183 negatively correlated with the cryocrit percentage in patients (P = 0.0052, data not shown). Interestingly, only memory CD86 levels were significantly elevated in cryo+ patients with a history of symptoms versus those cryo+ patients without symptoms (P = 0.0402). While there was a trend for increased expression of memory B cell HLA-DR in cryo+ patients with symptoms (P = 0.1597), all other markers were not significantly different, indicating that certain activation markers can be present before symptoms develop.

### Advanced Liver Fibrosis is Associated with Increased Memory B cell Expression of CD86 and Higher Serum AFP Levels

**Figure 6:**
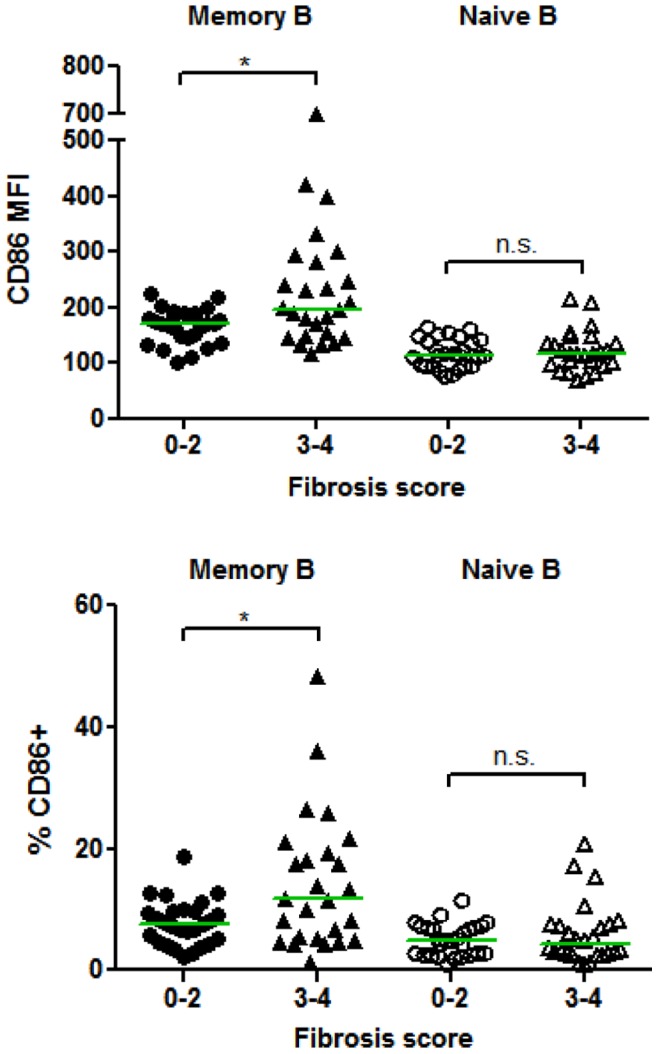
HCV patients with advanced fibrosis have increased expression of CD86 on memory B cells. Fibrosis scores were determined as outlined in the Materials and Methods, where scores of 3–4 are considered advanced fibrosis/cirrhosis (F4). PBMCs were freshly isolated and analyzed by flow cytometry as in Figure 3. The expression level and percent positive of cells expressing CD86 (geometric mean fluorescent intensity (MFI) or percent positive compared to mouse isotype controls) were calculated for memory (CD19+CD27+) and naïve (CD19+CD27−) B cells. Horizontal lines represent median values. *, P<0.05; n.s., not significant.

Expression of the CD86 activation marker on memory B cells was found to be significantly elevated in patients with advanced liver fibrosis (Figure 6; P = 0.0155). In addition, the percentage of memory B cells expressing CD86 was significantly elevated, but all other markers on memory B cells were not significantly changed (Figure 6; P = 0.032). Naïve B cells were not differentially activated in advanced fibrosis since the percentage of naïve B cells expressing CD86 and all other markers measured were not significantly different. There was also no significant difference in HCV serum titers, or total or memory B cell percentages. When we compared non-cirrhotic to cirrhotic patients, we found that HCV patients with cirrhosis have significantly lower percentages and numbers of memory, but not naïve B cells (P≤0.01), which is in agreement with Doi et al. [27]. The percent of CD5+ B1 B cells did not significantly differ between HCV patients with varying degrees of fibrosis (data not shown). Another marker that was significantly elevated in advanced liver fibrosis was alpha-fetoprotein (AFP) (P = 0.0019), which has previously been shown to be elevated in HCV patients with cirrhosis and is a clinical marker of HCC [28], [29], although none of our patients had been diagnosed with HCC at present. In our cohort, ALT levels were not significantly higher in patients with fibrosis scores of 3–4 (P = 0.2064).

## Discussion

The association of HCV with B cell lymphoproliferative diseases has been known for many years, but characterization of the phenotype of B cells in chronic HCV has been limited to studies of populations in Italy and the United States, with varying results. The genetic background of patients in Italy could partially account for why this population has a much higher incidence of cryoglobulinemia compared to other areas of the world (reviewed in [10]). Our study focused on a cohort of 54 chronic HCV infected patients in Alberta, Canada with varying degrees of liver disease. The specific goals of this study were to determine if B cells are activated in HCV patients compared to healthy controls, and whether activation was enhanced in patients with cryoglobulinemia or advanced liver disease. We found that the numbers of total B cells and memory and naïve subsets were not significantly changed in chronic HCV infected patients and that only memory B cells were significantly activated. For the first time, we found that patients with detectable cryoglobulins have elevated levels of memory B cell CD86, HLA-DR and CD71 compared to HCV patients without detectable cryoglobulins. Since only CD86 was associated with a history of symptoms, monitoring the expression of HLA-DR and CD71 could now be used to detect cryoglobulinemia prior to the onset of clinical disease. In addition, only naïve B cell CD183 expression levels negatively correlated with the percent cryocrit, which could indicate that naïve B cell migration is altered in those patients with the highest levels of cryoglobulins. Further work is needed to investigate the expression of other chemokine receptors such as CXCR5, which was shown to be negatively correlated with the expression of CD183 on rheumatoid arthritis patient’s B cells [30]. We also report for the first time that memory B cell CD86 expression levels and percentages of memory B cells expressing CD86 were significantly associated with advanced liver disease, whereas all other activation markers measured remained unchanged.

The most recent literature on B cell biology in HCV infection has focused on two main aspects: overall B cell analysis and re-activation *in vitro* and characterization of a clonal expansion of B cells specifically in cryoglobulinemia that produce rheumatoid factor. Our analysis concentrated on the status of B cell activation in chronic HCV infected patients and specifically focused on how this phenotype is altered in cryoglobulinemia and advanced liver fibrosis. Three recent studies have also examined the B cell activation status in chronic HCV patients. Ni et al. [18] found that B cells were not activated in HCV patients compared to healthy controls. While this was not replicated in our study or in two others, two reasons for this difference could be that first, cryopreserved PBMCs were analyzed and a later publication by Rosa et al. [20] demonstrated that cryopreservation downregulates activation markers on B cells. Secondly, only total CD19+ B cells were examined for activation markers in Ni et al. [18] meaning that changes in memory B cells may have gone undetected because the majority of B cells in peripheral blood are naïve.

The second recent study on B cells in HCV infected patients found elevated percentages of CD69+, CD71+, CD86+ and CD183+ naïve and memory B cells [20]. Opposite to our findings, they found more pronounced activation differences in the naïve B cell population and they found significantly higher percentages of memory B cells in HCV patients compared to healthy controls or patients with hepatitis B virus infection. Importantly, only patients who responded to treatment exhibited decreases in B cell activation. They found no difference in B cell activation between patients with or without cryoglobulinemia. We speculate that the differences between this report and our study could be due to differences in the patient population and/or the smaller number of control individuals included by Rosa et al. (n = 21) [20].

The third major study on B cell activation performed a similar analysis and found that naïve and memory B cells were activated in chronic HCV patients, but predominantly memory cells were activated similar to our findings [19]. Unlike in our analysis, they did not see correlations of B cell activation with liver disease markers (ALT, GGT), fibrosis or cryoglobulinemia, whereas our correlations were statistically significant. A strength of our analysis was that every HCV patient that came into clinic was tested for cryoglobulins, leading to 14 new diagnoses that may have been missed if we tested solely based on the presence of clinical cryoglobulinemia symptoms. Therefore, a main reason the studies above may have not found an association of B cell activation with cryoglobulins is that some patients with detectable cryoglobulins may have been included in a cryo- group, especially since patients in the Mediterranean area have the highest frequency of cryoglobulinemia in the world [10].

Approximately 10–15% of HCV patients will develop cirrhosis in the first 20 years of infection [2]. This rate is much higher in patients with cryoglobulinemia (up to 40%), but more studies are needed to study the relationship between these two manifestations of HCV infection. Doi et al. [27] found significantly decreased memory B cells when comparing patients with cirrhosis to patients with little to no fibrosis or healthy controls. We also found a significant decrease in the percent and number of memory B cells, but not naïve B cells in patients with cirrhosis (data not shown). This decrease in memory B cells could be due to cells migrating to the inflamed liver, because we and others have shown increased levels of CD183, which promotes migration to the liver [19], [20], [31]. Another possibility is that B cells undergo activation induced apoptosis, as we did find increased levels of the activation marker CD86 specifically on the memory B cell subset.

In conclusion, we have shown that memory B cells, and generally not naive B cells, are activated in chronic HCV infections and that the expression of memory B cell CD86 correlates with advanced liver fibrosis. Whether the activation phenotype is stable during HCV infection remains to be tested and in the future we will study a cohort of patients longitudinally and specifically look at memory B cell CD86 expression to see if increased expression predicts the development of fibrosis/cirrhosis. In addition, we have demonstrated that certain memory B cell activation markers (CD86, CD71 and HLA-DR) correlate with the development of HCV-associated cryoglobulinemia with CD71 and HLA-DR levels increasing independently of the presence of clinical symptoms. Apart from being of potential diagnostic significance, these activation phenomenon add to our knowledge of this complex disease. Future work will be directed at understanding the underlying mechanisms of these activation processes using *in vitro* B cell cultures, especially for whether HCV itself or the envelope glycoprotein E2 upregulates the markers we have identified, and what the role of these activation markers are in the development of HCV-associated cryoglobulinemia and B cell non-Hodgkin lymphoma.
